# *Coxiella burnetii* Seroprevalence and Risk Factors in Cattle Farmers and Farm Residents in Three Northeastern Provinces and Inner Mongolia Autonomous Region, China

**DOI:** 10.1155/2016/7059196

**Published:** 2016-02-04

**Authors:** Wu-Wen Sun, Wei Cong, Mao-Hui Li, Chun-Feng Wang, Xiao-Feng Shan, Ai-Dong Qian

**Affiliations:** College of Animal Science and Technology, Jilin Agriculture University, Changchun, Jilin Province 130118, China

## Abstract

Little is known about* Coxiella burnetii *infection among cattle farmers and farm residents in China. Thus, the present study was conducted to detect the seroprevalence of* C. burnetii *infection and estimate associated risk factors among cattle farmers and farm residents in China. A cross-sectional study was designed, and sera of 362 people living or working on 106 cattle farms were tested for* C. burnetii* IgG and IgM antibodies by immunofluorescence assay. Overall* C. burnetii* seroprevalence was 35.6% (129/362, 95% CI: 30.70–40.57), and 112 participants had experienced a past infection and seventeen (4.7%) had experienced a relatively recent infection. In the final combined multilevel model, the following activities were significantly associated with presence of antibodies against* C. burnetii*: milking cattle, providing general healthcare to cattle, providing birth assistance, contact dead-born animals, urbanization, and presence of mice and/or rats in the stable. Moreover, presence of disinfection equipment was a significant protective factor. This is the first study addressing the seroprevalence and risk factors of* C. burnetii *infection in cattle farmers and farm residents in three northeastern provinces and Inner Mongolia Autonomous Region, China.

## 1. Introduction

Q fever, caused by* Coxiella burnetii* (*C. burnetii*), is a ubiquitous zoonotic disease. Cattle, sheep, and goats are considered as the primary animal reservoirs for human infection.* C. burnetii* are shed in particularly high concentrations in placentas and birth fluids of infected animals, which may subsequently contaminate the stable environment [1]. Human get infections with* C. burnetii* mostly by inhalation of contaminated aerosols coming from parturient animals and their birth products [2–4]. Clinical symptoms of acute Q fever usually present as a self-limited febrile illness, hepatitis, or pneumonia, with very little proportion evolving into chronic infections [5–7].

Q fever has outbroken in people in some countries, including Spain [8], Switzerland [9], Great Britain [10], Germany [11], and Netherlands [12]. Infections are usual occupational risk in persons working with livestock and contacting with highly infectious aerosols from birth products, milk, urine, faeces, or semen of infected animals [13]. These occupational risk populations include workers in slaughterhouses, meat-packing plants, and tanneries as well as veterinarians and farmers [13]. In China, infection has been detected in humans as well as in a wide range of wild, domestic, and farmed animals such as cattle, goats, dogs, pigs, mice, sheep, and horses [14]. In the previous study, we reported the seroprevalence of* C. burnetii* infection in farmed ruminants including cattle in the three northeastern provinces and Inner Mongolia Autonomous Region, China [15]. However, information on the seroprevalence and risk factors for acquisition of* C. burnetii* infection in cattle farmers and farm residents is limited. Thus, the aim of the present study was to determine the seroprevalence in farmers and household members living and/or working on cattle farms and to assess the farm-related and individual risk factors for seropositivity in order to update control measures and to provide targeted advice for this occupational group and the China cattle industry.

## 2. Materials and Method

### 2.1. Study Population and Data Collection

This study was approved by the Animal Ethics Committee of Jilin Agriculture University, China. All cattle farms in three northeastern provinces and Inner Mongolia Autonomous Region with at least 50 cattle that were not vaccinated for Q-fever were selected from the register in the census of the zone. As an important cattle and sheep breeding base in China, with the development of economy, farms with different sizes were settled up quickly in Inner Mongolia Autonomous Region. The three northeastern provinces (Jilin, Liaoning, and Heilongjiang provinces) are comprehensive agricultural bases. Poultry, pigs, cattle, sheep, and deer are the main breeding animals in these areas. On eligible farms, we approached cattle farmers and one or two of their household members aged 12 years and older, and in some cases, other persons working or living on the farm such as farm employees. A maximum of five participants were included per farm. Nonresponders received a reminder 3 weeks after the initial invitation. After providing informed consent on farm and individual level, all participating farms were visited by professional laboratory assistants, who collected sera from October 2013 through July 2014. Each participant completed a questionnaire about personal characteristics (e.g., age, medical history, farm-related activities, contact with livestock and companion animals, and use of personal protective equipment). The farm owner or manager completed a questionnaire about herd size, cattle housing, presence of other livestock and companion animals, farm facilities, and hygiene measures.

### 2.2. Serological Method

An immunofluorescence assay (IFA) (Focus Diagnostics, Cypress, CA, USA) was used to test serum samples for* C. burnetii* phases I and II IgM and IgG. All samples were screened at an initial dilution of 1 : 32; those with negative results were considered negative. Positive samples were further classified as indicative of relatively recent infections (IgM phase II titer >32) or past infections (IgG phase II titer >32 and IgM phase II titer <32). Samples with all other outcomes were considered negative. The term relatively recent was chosen because phase II IgM is commonly found up to 1 year after infection in acute Q fever cases, but it may persist up to 3 years [16]. Phases I and II IgG end point titers were determined for all seropositive persons. In agreement with chronic Q fever diagnostic criteria used in the Netherlands [17], phase I IgG titers ≥1,024 in samples in the past infection group were considered indicative of possible chronic infection.

### 2.3. Statistical Analysis

Results were analyzed with SPSS 19.0 software package. For comparison of the frequencies among the groups, the Mantel-Haenszel test and when indicated the Fisher exact test were used. Bivariate, multivariate, and multilevel analyses were used to assess the association between participant- and farm-based characteristics of the subjects and the* C. burnetii* infection. Variables were included in the multivariate analysis if they had a *P* value of equal or less than 0.20 in the bivariate analysis. Adjusted odd ratio (OR) and 95% confidence interval were calculated by multivariate analysis using multiple, unconditional, and logistic regression. A *P* value less than 0.05 was considered statistically significant.

## 3. Results

### 3.1. Descriptive Characteristics

Of all 197 invited eligible cattle farms, 106 (53.8%) farms participated in this study. The number of cattle farms from Heilongjiang, Jilin, Liaoning, and Inner Mongolia was 21, 28, 22, and 35, respectively. The mean herd size was 95 cattle (range 50–327) in participating farms. From the 106 participating farms, 362 persons provided a blood sample (mean age 46.0 years (12–68), 45.3% male) (Table 1). All of the farm-based and participant-based questionnaires were completed by the 106 farmers and 362 persons.

### 3.2. Seroprevalence of* C. burnetii*

Overall* C. burnetii* seroprevalence was 35.6% (129/362, 95% CI: 30.70–40.57), and seroprevalence among farmers, spouses, children, and others was 38.3%, 31.5%, 31.1%, and 37.1%, respectively (Table 1). Of the 129 seropositive participants, 112 participants had experienced a past infection and seventeen (4.7%) had experienced a relatively recent infection, as demonstrated by presence of IgM phase II antibodies. IgG phase II end titers were known for the 76 participants with a past infection with IgG phase I <1 : 32: 1 : 32 (*n* = 25), 1 : 64 (*n* = 14), 1 : 128 (*n* = 13), 1 : 256 (*n* = 13), 1 : 512 (*n* = 10), and ≥1 : 1024 (*n* = 1). For the 36 participants with a past infection with IgG phase I ≥1 : 32, 3 persons had “possible chronic Q fever” with IgG phase I titers ≥1 : 1024 according to diagnostic standard used in the Netherlands [8]. We could not confirm that these truly were chronic Q fever cases due to lack of clinical information (e.g., presence of vascular infection, endocardial involvement, or other clinical risk factors).

### 3.3. Risk Factors for* C. burnetii* Infection

All individual and farm-based variables, which were tested in the bivariate analysis for relationship with human* C. burnetii* seropositivity, are shown in Tables 2 and 3.

In the multivariate analyses, from 14 individual variables which were associated in the bivariate analysis, six were independently associated with* C. burnetii* seropositivity (Table 4). Moreover, 5/18 farm-based variables included in the multilevel analyses were significantly independent risk or protective factors and together were used as the full multilevel start model (Table 5).

In the final combined multilevel model, significant risk factors were milking cattle, general healthcare of cattle, birth assistance, contact dead-born animals, urbanization, and presence of mice and/or rats in the stable. Moreover, presence of disinfection equipment was a significant protective factor (Table 6).

## 4. Discussion

This is the first study exploring the seroprevalence in cattle farmers and farm residents in China, and one of few risk factor studies on human* C. burnetii* infections in farm populations worldwide [18–20], suggesting that living and or working on cattle farm has a high lifetime risk for acquiring* C. burnetii* infection. Farmers and other household members are usually at highest risk for acquiring* C. burnetii* infection due to close contact with infected cattle and contaminated stables on farms.

The detected seroprevalence was high not only for the farmers (38.3%), as expected, but also among spouses (31.5%), children (31.1%), and others (37.1%) who lived and often also worked at the farm. The present seroprevalence obviously overs the estimates of 10.2% in the people studied to date in China [14]. The seroprevalence was also lower than those in other studies focusing on, nonfurther specified, farm populations, such as 49% among farmers from Northern Ireland [21], 72.1% in dairy cattle farmers in the Netherlands [18], but was comparable to the 27% seroprevalence in a farm cohort in the United Kingdom [10]. However, it is complicated to compare these seroprevalences due to some differences, including geographical conditions, the different study populations, diagnostic methods, and living styles. Moreover, in the present study, females have a higher seroprevalence than males. It is contrary to other places in the world where studies have suggested that males are more susceptible to* C. burnetii* infection [2]. Routine activities of women in rural areas including taking care of livestock and cleaning stables result in the high prevalence in females in China [14].

Several independent individual and farm-based risk factors for* C. burnetii* seropositivity were found such as working and/or living in farm, milking cattle, general healthcare of cattle, birth assistance, contacting raw milk, contacting cattle manure, contacting dead-born animals, urbanization, laying hens on farm, use of silage, presence of mice and/or rats in the stable, presence of disinfection equipment, and birds in stable. The individual risk factor involving direct contact with cattle or dust-producing activities in the cattle stable, such as milking, general healthcare of cattle, clean stables, birth assistance, contacting raw milk, contacting cattle manure, and contacting dead-born animals, reflects the stable environment contact [22]. Under these circumstances the risk of inhalation of contaminated aerosols is high, with a potential increased risk for acquiring an infection. The degree of total farm animal contact has been reported to seem more important than particular animal exposure, suggesting that risk of* C. burnetii* exposure is largely connected with farm environment contact [6, 22].

Two farm-related risk factors were identified to be associated with human seropositivity among cattle farm residents/staff: urbanization and presence of mice and/or rats in the stable. The concentration and management of cattle farming in the rural area of the study regions possibly promoted transmission between farms. The presence of mice and/or rats in the stable was observed as risk factor for human seropositivity, suggesting* C. burnetii* introduction or facilitation of spread by infected wild animals [23–25]. Moreover, presence of disinfection equipment was observed as protective factor for human seropositivity.

It is worth noting that Q fever is out of notifiable diseases in China and thus it is not easy to get test facilities. Most cases are diagnosed through retrospective and epidemiological studies which implies that misdiagnosis often occurred for acute cases, resulting in the greater possibility of chronic infections which have a poor prognosis and high mortality [14]. Thus, routine serological follow-up is helpful for prevention as well as basic biological safety rules, such as hygiene measures and the use of protection clothes.

To conclude, high* C. burnetii* seroprevalences demonstrate that cattle farmers and farm residents have a substantial lifetime risk for acquiring this zoonotic infection. We recommend reinforcing routine biosecurity measures to avoid indirect spread, avoiding access of companion and wild animals to the stable, and offer advice on eliminating nuisance animals in the cattle stables. Clinicians should strengthen their awareness to consider Q fever in this occupational group presenting with compatible symptoms or relevant sequelae to allow diagnosis and therapy in time.

## Figures and Tables

**Table 1:** Participant characteristics and *Coxiella burnetii* seroprevalence among cattle farmers and farm residents in three northeastern provinces and Inner Mongolia Autonomous Region, China.

Variable	Category	Freq. (*N*)	Seroprevalence (%)	95% CI
Participant		362	35.64	30.70–40.57

Sex	Male	198	36.36	29.66–43.06
	Female	164	34.76	27.47–42.04

Age group	<35	56	35.71	23.16–48.26
	35–44	78	30.77	20.53–41.01
	45–54	137	38.69	30.53–46.84
	≥55	91	35.17	25.35–44.98

Function	Farmer	193	38.34	31.48–45.20
	Spouse	89	31.46	21.81–41.11
	Child	45	31.11	17.59–44.64
	Other^*∗*^	35	37.14	21.14–53.15

Region	Heilongjiang	98	30.61	21.49–39.74
	Jilin	76	27.63	17.58–37.69
	Liaoning	92	31.52	22.03–41.02
	Inner Mongolia	96	51.04	41.04–61.04

*∗* represents other family members and employees.

**Table 2:** Bivariate logistic regression analysis of participant-based characteristics associated with *Coxiella burnetii* positivity among cattle farmers and farm residents in three northeastern provinces and Inner Mongolia Autonomous Region, China.

Variable	Category	Freq. (*N*)	Seroprevalence (%)	OR (95% CI)	*P* value
Work and/or live on farm	Work and live	245	40.41	2.03 (0.98–4.22)	0.023
	Work, but not live	73	26.03	1.06 (0.45–2.49)	
	Not working, but live	44	25.00	Reference	

Hours working on farm	Fulltime	174	37.36	1.32 (0.65–2.66)	0.853
	Halftime	109	35.78	1.23 (0.59–2.59)	
	Quarter week	34	32.35	1.06 (0.41–2.76)	
	Sometimes/never	45	31.11	Reference	

How often in stable	Every day	189	38.62	1.39 (0.70–2.79)	0.426
	Every week	104	35.58	1.22 (0.58–2.58)	
	Every month	38	28.95	0.90 (0.35–2.32)	
	Less than once a month/never	31	25.81	Reference	

Feeding cattle	Yes	299	38.45	2.19 (1.16–4.14)	0.014
	No	63	22.22	Reference	

Milking cattle	Yes	203	43.84	2.32 (1.48–3.65)	<0.001
	No	159	25.16	Reference	

General healthcare of cattle	Yes	241	42.32	2.56 (1.55–4.21)	<0.001
	No	121	22.31	Reference	

Remove manure	Yes	286	35.66	1.01 (0.59–1.71)	0.982
	No	76	35.53	Reference	

Spread manure	Yes	258	40.31	2.13 (1.28–3.57)	0.002
	No	104	24.04	Reference	

Clean stables	Yes	269	39.03	1.84 (1.09–3.11)	0.022
	No	93	25.81	Reference	

Birth assistance	Yes	216	42.59	2.19 (1.38–3.46)	<0.001
	No	146	25.34	Reference	

Administration	Yes	219	38.81	1.43 (0.91–2.23)	0.076
	No	143	30.77	Reference	

Wear overalls or boots	Yes	233	38.63	1.45 (0.92–2.30)	0.110
	No	129	30.23	Reference	

Having a dog	Yes	294	35.03	0.87 (0.51–1.50)	0.619
	No	68	38.24	Reference	

Having a cat	Yes	278	35.25	0.93 (0.56–1.55)	0.782
	No	84	36.90	Reference	

Direct contact with cattle in their own or other farms	Yes	287	38.68	2.00 (1.12–3.57)	0.018
	No	75	24.00	Reference	

Direct contact with horses in their own or other farms	Yes	210	39.52	2.56 (1.61–4.07)	<0.001
	No	152	23.68	Reference	

Contact with raw milk	Yes	243	43.21	3.01 (1.80–5.04)	<0.001
	No	119	20.17	Reference	

Contact with cattle manure	Yes	225	44.00	2.80 (1.73–4.54)	<0.001
	No	137	21.90	Reference	

Contact with dead-born animals	Yes	196	45.41	2.62 (1.67–4.12)	<0.001
	No	166	24.10	Reference	

Contact with placenta/birth material	Yes	188	38.30	1.27 (0.83–1.96)	0.272
	No	174	32.76	Reference	

**Table 3:** Bivariate logistic regression analysis of farm-based characteristics associated with *Coxiella burnetii* positivity among cattle farmers and farm residents in three northeastern provinces and Inner Mongolia Autonomous Region, China.

Variable	Category	Number of humans tested	Positive (%)	OR (95% CI)	*P* value
Region	Heilongjiang	98	30.61	Reference	
	Jilin	76	27.63	0.87 (0.45–1.68)	0.667
	Liaoning	92	31.52	1.04 (0.56–1.93)	0.892
	Inner Mongolia	96	51.04	2.36 (1.31–4.25)	0.004

Urbanization	Moderate or minor urban area	149	20.81	Reference	<0.001
	Rural area	213	46.01	3.24 (2.01–5.24)	

Herd size	Small (50–100)	197	32.48	Reference	0.393
	Medium (100–150)	104	39.42	1.35 (0.83–2.22)	
	Large (>150)	61	39.34	1.35 (0.74–2.44)	

Beef cattle on the farm	Yes	202	37.13	1.16 (0.75–1.79)	0.505
	No	160	33.75	Reference	

Number of stables	>3 stables	117	32.48	0.81 (0.51–1.30)	0.386
	≤3 stables	245	37.14	Reference	

Use of artificial insemination	Yes	84	46.43	1.81 (1.10–2.98)	0.018
	No	278	32.37	Reference	

Laying hens on farm	Yes	243	40.33	1.92 (1.18–3.11)	0.008
	No	119	26.05	Reference	

Presence of cat(s) in cattle stable	Present	198	36.36	1.07 (0.70–1.65)	0.751
	Absent	164	34.76	Reference	

Use of silage	Yes	276	40.22	2.54 (1.43–4.51)	0.001
	No	86	20.93	Reference	

Use of maize	Yes	288	38.19	1.79 (1.01–3.17)	0.045
	No	74	25.68	Reference	

Feeding method	Fodder mixer or automatic	241	32.37	0.66 (0.42–1.03)	0.067
	Hand/wheelbarrow	121	42.15	Reference	

Presence of mice and/or rats in the stable	Present	199	45.23	2.63 (1.67–4.14)	<0.001
	Absent	163	23.93	Reference	

Farm visitors	Yes	143	37.76	1.17 (0.75–1.81)	0.495
	No	219	34.25	Reference	

Farm boots for staff	Yes	277	36.46	1.17 (0.70–1.95)	0.553
	No	85	32.94	Reference	

Presence of hygienic locker room	Yes	269	31.97	0.55 (0.34–0.88)	0.013
	No	93	46.24	Reference	

Presence of disinfection equipment	Yes	224	29.46	0.50 (0.32–0.77)	0.002
	No	138	45.65	Reference	

Birds in stable	Yes	90	45.56	1.75 (1.08–2.85)	0.023
	No	272	32.35	Reference	

Veterinary service	Yes	301	31.62	1.40 (0.78–2.54)	0.273
	No	61	29.51	Reference	

Type of farm management	Closed herd	298	34.89	0.84 (0.48–1.46)	0.528
	Purchase of cattle	64	39.06	Reference	

**Table 4:** Multivariate logistic regression analysis of participant-based characteristics associated with *Coxiella burnetii* positivity among cattle farmers and farm residents in three northeastern provinces and Inner Mongolia Autonomous Region, China.

Variable^a^	Category	Adjusted odds ratio^b^	95% confidence interval	*P* value
Milking cattle	Yes	1.88	1.21–2.94	0.005
	No	Reference		

General healthcare of cattle	Yes	2.40	1.46–3.93	<0.001
	No	Reference		

Birth assistance	Yes	2.07	1.31–3.27	0.002
	No	Reference		

Contact raw milk	Yes	2.47	1.50–4.06	<0.001
	No	Reference		

Contact cattle manure	Yes	3.23	1.92–5.44	<0.001
	No	Reference		

Contact dead-born animals	Yes	3.45	2.16–5.50	<0.001
	No	Reference		

^a^The variables included were those with a *P* ≤ 0.20 obtained in the bivariate analysis.

^b^Adjusted by age and the rest of characteristics included in this table.

**Table 5:** Multivariate logistic regression analysis of farm-based characteristics associated with *Coxiella burnetii* positivity among cattle farmers and farm residents in three northeastern provinces and Inner Mongolia Autonomous Region, China.

Variable^a^	Category	Adjusted odds ratio^b^	95% confidence interval	*P* value
Urbanization	Rural area	3.66	2.25–5.96	<0.001
	Moderate or minor urban area	Reference		

Laying hens on farm	Yes	2.04	1.25–3.32	0.004
	No	Reference		

Use of silage	Yes	1.99	1.15–3.44	0.013
	No	Reference		

Presence of mice and/or rats in the stable	Present	2.49	1.58–3.91	<0.001
	Absent	Reference		

Presence of disinfection equipment	Yes	0.58	0.37–0.90	0.015
	No	Reference		

^a^The variables included were those with a *P* ≤ 0.20 obtained in the bivariate analysis.

^b^Adjusted by age and the rest of characteristics included in this table.

**Table 6:** Combined multilevel analysis of participant- and farm-based characteristics associated with *Coxiella burnetii* seropositivity among cattle farmers and farm residents in three northeastern provinces and Inner Mongolia Autonomous Region, China.

Variable^a^	Category	Adjusted odds ratio^b^	95% confidence interval	*P* value
Milking cattle	Yes	2.09	1.33–3.27	0.001
	No	Reference		

General healthcare of cattle	Yes	2.25	1.38–3.67	0.001
	No	Reference		

Birth assistance	Yes	1.86	1.18–2.92	0.005
	No	Reference		

Contact dead-born animals	Yes	2.67	1.69–4.45	<0.001
	No	Reference		

Urbanization	Rural area	2.34	1.41–3.45	<0.001
	Moderate or minor urban area	Reference		

Presence of mice and/or rats in the stable	Present	1.95	1.38–3.01	0.002
	Absent	Reference		

Presence of disinfection equipment	Yes	0.37	0.20–0.49	<0.001
	No	Reference		

^a^The variables included were those with a *P* ≤ 0.20 obtained in the bivariate analysis.

^b^Adjusted by age and the rest of characteristics included in this table.
